# Decomposition of Cyclohexane on Ni_3_Al Thin Foil Intermetallic Catalyst

**DOI:** 10.3390/ma7107039

**Published:** 2014-10-17

**Authors:** Paweł Jóźwik, Marco Salerno, Wojciech J. Stępniowski, Zbigniew Bojar, Krzysztof Krawczyk

**Affiliations:** 1 Department of Advanced Materials and Technologies, Faculty of Advanced Technologies and Chemistry, Military University of Technology, Kaliskiego 2 Str., 00-908 Warszawa, Poland; E-Mails: pjozwik@wat.edu.pl (P.J.); wstepniowski@wat.edu.pl (W.J.S.); zbojar@wat.edu.pl (Z.B.); 2 Department of Nanophysics, Istituto Italiano di Tecnologia, via Morego 30, I-16163 Genova, Italy; 3 Faculty of Chemistry, Warsaw University of Technology, Noakowskiego 3, 00-664 Warszawa, Poland; E-Mail: kkrawczyk@wat.edu.pl

**Keywords:** nickel aluminides, Ni_3_Al intermetallic alloy, catalysis, cyclohexane

## Abstract

Micro-grained thin foils made of Ni_3_Al intermetallic alloy were fabricated, according to a previously described procedure, and tested as catalyst for decomposition of cyclohexane. The conversion efficiency of the catalyst was evaluated in a synthetic air atmosphere, and found to be as high as 98.7% ± 1.0% at 600 °C and 86.7% ± 3.6% at 500 °C. During the reaction, the growth of carbon nanofibers on the catalysts surface was observed. The chemical and phase composition of the nanofibers was investigated with scanning electron microscopy (SEM), energy dispersive spectrometry (EDS) and X-ray diffraction (XRD), finding them to be made of graphitic carbon. Additionally, nanoparticles of nickel appear to be incorporated in the fibers. The obtained material is promising for large scale fabrication in industrial applications because of its high efficiency in the hydrocarbon decomposition, the simple fabrication procedure, and the form of self-supporting foils with the presence of additional carbon nanofibers that increase its efficiency.

## 1. Introduction

Intermetallic Ni_3_Al-based alloys are a group of advanced materials which show potential outstanding physical and chemical properties, such as excellent oxidation and corrosion resistance, fairly high melting temperatures, relatively low densities and anomalous strengthening with increasing temperature. Extensive efforts have been made to develop Ni_3_Al-based alloys into commercial use as high-temperature structural material in a bulk form, e.g., as-cast ingots, bars, plates, tubes, *etc.* [1,2,3,4].

Thin foils made of Ni_3_Al intermetallic alloy can be used in high-performance applications in the form of honeycomb structures, which have the advantage of being lightweight and showing high-stiffness and catalytic properties [4,5,6,7]. In previous work, a cold‑rolling based approach was set up which allows to obtain micro- or nano-structured Ni_3_Al intermetallic foils as thin as 50 µm thickness [8]. Another method to obtain similar thin foils-only with microcrystalline structure-was reported by Hirano *et al.* [9,10], who rolled the directionally solidified ingots at room temperature. The mechanical properties of so-prepared Ni_3_Al materials were studied in detail [9,10,11]. The materials obtained with this method have good catalytic properties for alcohols and hydrocarbons decomposition [5,6,12].

The present paper focuses on the catalytic decomposition of cyclohexane on Ni_3_Al intermetallic alloy thin foils. The performance of the catalyst, represented by conversion and its surface morphology changes, has been systematically investigated.

## 2. Results and Discussion

**Figure 1:**
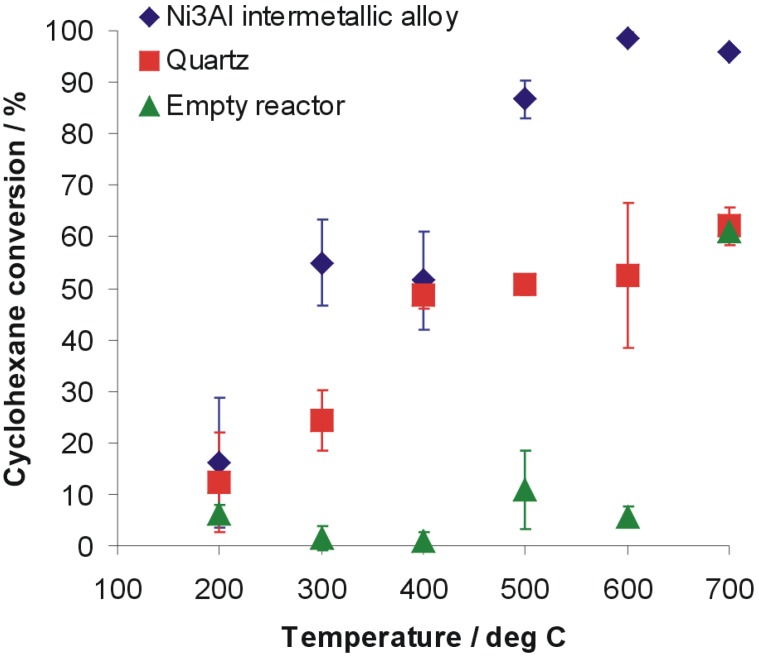
Conversion of cyclohexane conducted on the pieces of Ni_3_Al thin foil, quartz and empty reactor as a function of the temperature.

Figure 1 shows cyclohexane conversion *versus* reaction temperature for Ni_3_Al intermetallic alloy and quartz as a reference inert material and additionally by using empty reactor. The cyclohexane conversion on the pieces of Ni_3_Al foil with increasing temperature is generally fast and starts already above 200 °C. Since 500 °C the conversion efficiency is 86.7% ± 3.6%, while the conversion efficiency of quartz is approximately 50.6% ± 1.9%. Analysis of cyclohexane presence after examination with empty reactor revealed a very low level of conversion (approximately 10% or below) up to a temperature of 600 °C. Increasing the temperature above that level leads to growing efficiency of cyclohexane decomposition in the empty reactor up to 61.1% ± 1.0%, a level comparable to the highest conversion obtained for decomposition reaction conducted on quartz.

A major advantage of the applied catalyst is its simple form, pieces of thin foil without any support, which makes this material cost‑effective despite the significant content of nickel. Additionally, no complex, expensive and sophisticated chemistry is applied to form the catalyst and this approach allows its formation in industrial quantities. Moreover, the catalyst performance is comparable to, or even better than, those obtained with sophisticated chemical methods. For example, with Ni_2_P catalyst a conversion of cyclohexane close to 100% was achieved for similar temperatures, however in this case Al_2_O_3_ was applied as the support for the active material [13]. Application of nanoporous materials as a support for the catalyst, *i.e.*, Au@TiO_2_ also brings satisfactory catalytic results (approximately 10% of conversion at 150 °C). However, also in that case the catalyst manufacturing requires several steps [14].

**Figure 2:**
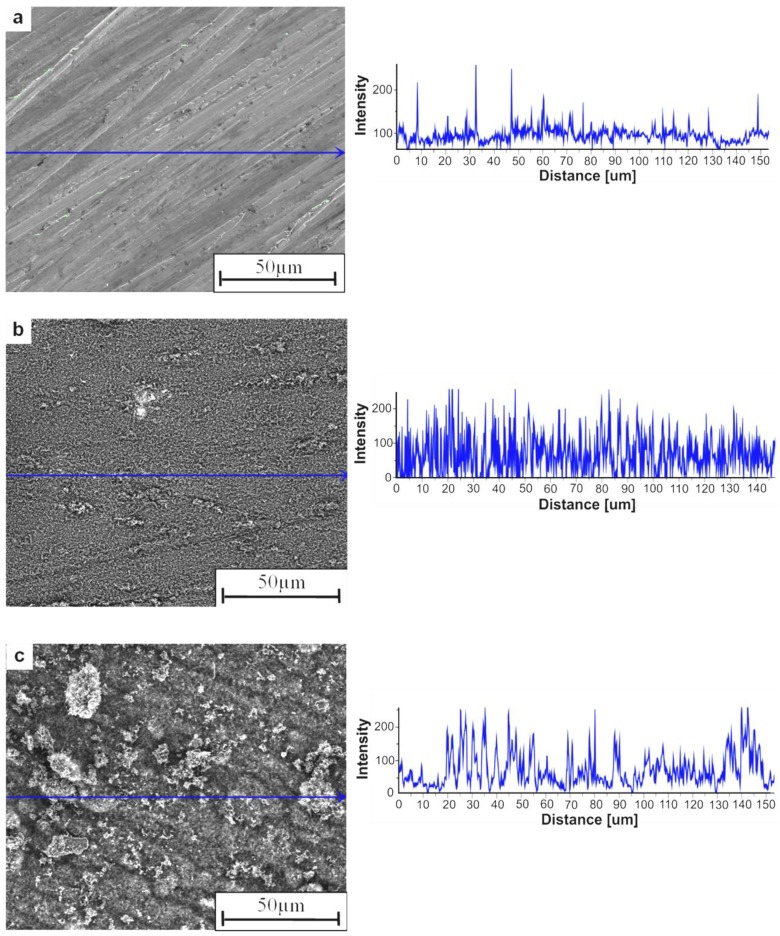
SEM morphology with intensity profiles of the surface of Ni_3_Al foil: (**a**) before experiments; and after decomposition of cyclohexane at: (**b**) 500 °C; (**c**) 700 °C.

An additional advantage of using the Ni_3_Al intermetallic alloy for hydrocarbons thermocatalytic decomposition is the formation of complex carbon nanostructures on the alloys surface (see Figure 2). It has been reported that carbon nanostructures are outstanding for hydrocarbons, *i.e.*, cyclohexane decomposition [15]. Typically, catalysts are being doped by the products of the reaction. In contrast, the products of the reaction—carbon nanostructures—also work as the catalyst with increased active surface area. Therefore, the catalytic performance of the Ni_3_Al intermetallic alloy is being enhanced by the reaction products, which is another advantage of this material.

**Figure 3:**
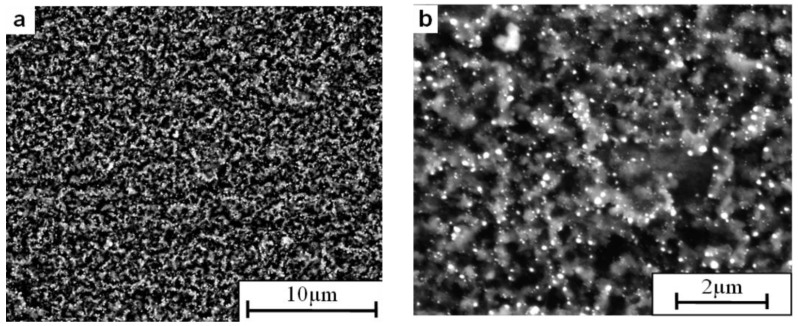
SEM morphology of carbon nanofibres (CNFs) deposited on the surface of Ni_3_Al foil after decomposition of cyclohexane at 500 °C, obtained by using a backscattered electrons (BSE) detector.

**Figure 4:**
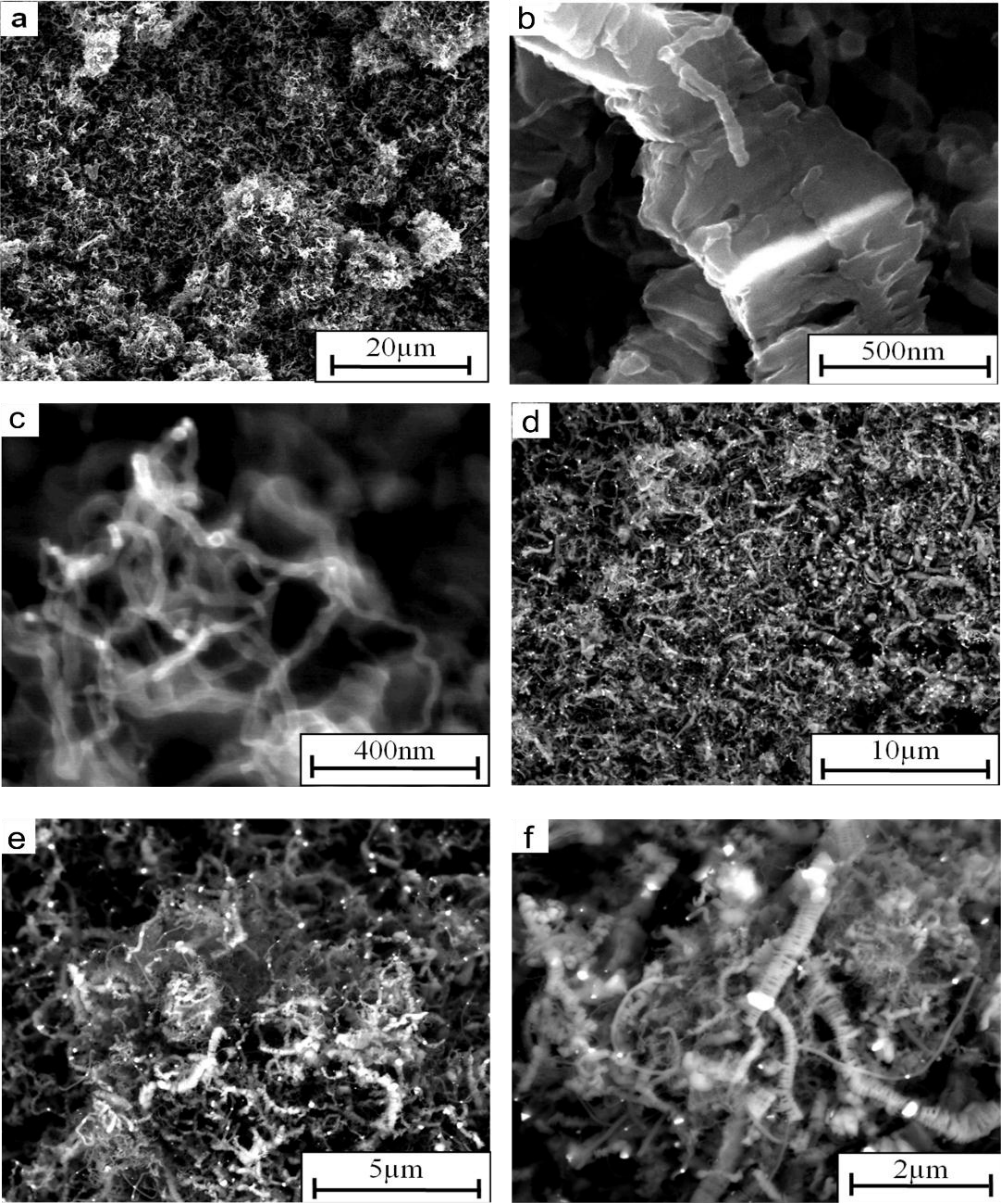
SEM morphology of CNFs deposited on the surface of Ni_3_Al foil after decomposition of cyclohexane at 700 °C, obtained by using (**a**–**c**) SE and (**d**–**f**) BSE detectors.

The pieces of Ni_3_Al thin foil, which have clean and glossy surfaces before the experiment, are covered with a deposit that looks like carbon black after the catalytic examination (Figure 2). The analysis of SEM morphology and intensity profiles of the Ni_3_Al foils surface before and decomposition of cyclohexane at 500 °C, shows the morphology developed for the deposit (Figure 2a,b). Further temperature increase up to 700 °C leads to the formation of clusters of carbon nanofibers (CNFs), which can be seen as a decrease in the intensity profiles (Figure 2c, Figure 3 and Figure 4). Observation of the Ni_3_Al foil by secondary electrons (SE) and backscattered electrons (BSE) signals in SEM, shows the presence of a complex structure of carbon nanofibers (CNFs) with various shapes and diameters, as well as metal-like contrast nanoparticles, deposited during cyclohexane decomposition (Figure 2 and Figure 3). The diffusion of carbon atoms through the catalyst centers leads to formation of tubular and platelet fibers on the foil surface. In the deposit, the tubular CNFs are dominant, with graphene layers rolled‑up in a cylindrical shape [16] (with average diameter below 80 nm) and bearing the metal‑like particles at the tip (see Figure 4c,d,f). In these CNFs, only a single face is available for precipitating carbon in the form of concentric graphite [17].

As the second component of the deposit, thicker platelet CNFs are observed, with an average diameter of approximately 500 nm [18]. As reported by Rodriguez *et al.* [17] and Martin-Gullon *et al.* [18], the catalyst particles are located inside the fiber (see Figure 4e), where carbon was formed by bidirectional mode. The platelet CNFs were formed simultaneously from opposite faces of the particle, which remains within the structure throughout the growth process [17].

**Figure 5:**
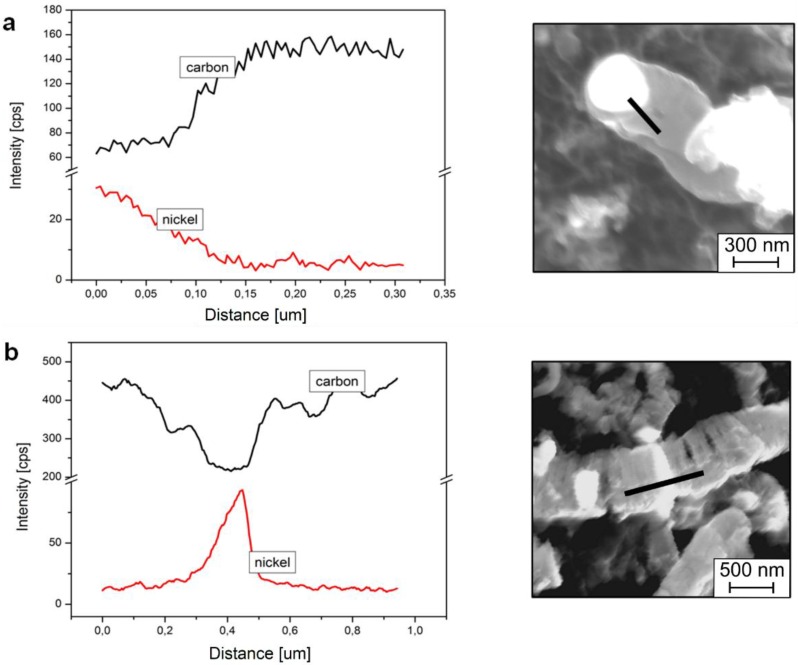
The energy dispersive spectrometry (EDS) linear analysis along the nanofiber with nickel‑like nanoparticles in (**a**) tubular and (**b**) platelet CNF.

In the case of the above-mentioned small metal-like objects, the result of EDS has only qualitative significance, but it is clear that the interior of the tubular CNFs is also rich in nickel and depleted from carbon (see metal nanoparticle in Figure 3b, Figure 4c–f and Figure 5b).

**Figure 6:**
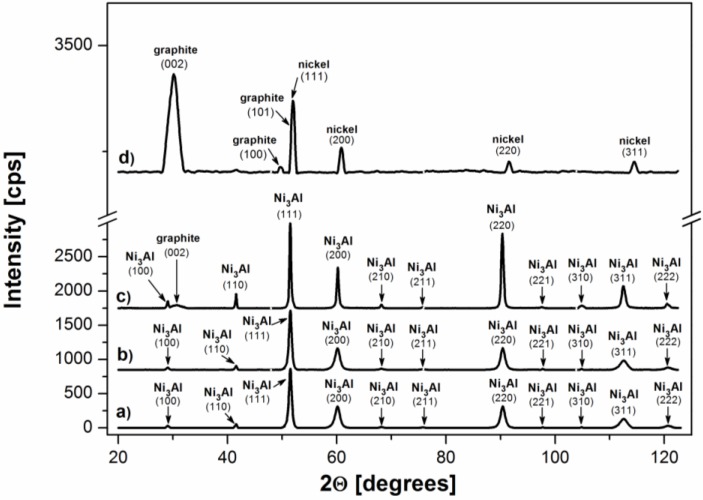
XRD patterns of Ni_3_Al foil surface: (**a**) before experiments; after cyclohexane decomposition at: (**b**) 500 °C and (**c**) 700 °C as well as deposit scraped off of the surface of Ni3Al foils (**d**).

The XRD patterns of the examined foils surface after cyclohexane decomposition at 700 °C (Figure 6a) show existence of Ni_3_Al intermetallic compound with slightly visible peak of graphite only from (002) plane, but without any apparent nickel contribution. The XRD analysis of Ni_3_Al foil after experiment at 500 °C shows only peaks characteristic for Ni_3_Al phase. The lack of nickel lattice is connected with similar *fcc*-based crystal structure to Ni_3_Al-phase and strong coincidence in their lattice constants (3.524 Å and 3.567 Å, respectively).

The XRD patterns for deposit after cyclohexane decomposition at 700 °C scraped from the Ni_3_Al foil (Figure 6d) demonstrate that the catalytic reaction products contain only pure nickel and graphite. The small nickel particles, probably formed in accordance with the mechanism described in previous reports [16,17,18], serve as an effective catalyst for cyclohexane decomposition, as well as CNFs, which are expected in common catalyst supports due to their large edges.

## 3. Materials and Methods

A Ni_3_Al intermetallic alloy with nominal composition of Ni–22.1Al–0.26Zr–0.1B (at%) was obtained by casting into shell-mold from pure elements melted in Ar atmosphere. The obtained ingots were cut into plates, cold-rolled up to 95% in few passes and recrystallized at 1100 °C for 1.5 h in argon atmosphere. As a result, 50 µm thick microcrystalline foil was obtained. More detailed procedures have been described previously [8]. Before catalytic experiments, the Ni_3_Al foil was mechanically polished and rinsed with acetone in an ultrasonic cleaner.

Catalytic decomposition of cyclohexane was carried out in a fixed bed quartz reactor preceded by preliminary quartz reactor where cyclohexane and air were being added and heated up. The investigations were conducted in a synthetic air atmosphere, with a composition of 21% O_2_, 10 ppm H_2_O or less in N_2_. The feed of the air and cyclohexane were set as 3 dm^3^/h by electronic flow controller and 3 mL/h of liquid hydrocarbon with infusion pump, respectively.

The catalytic activity of the Ni_3_Al intermetallic alloy was examined in the temperature range from 200 to 700 °C for 2 h. The gaseous products were analyzed online by the Clarus 500 gas-chromatograph equipped with Clarus 560S mass spectrometer (Perkin Elmer, Waltham, MA, USA). Small pieces of quartz were applied as the reference inert material. For all catalytic test the Ni_3_Al thin foil was cut into small pieces (approximately 2 × 3 mm^2^ each) and the total weight of quartz reactor feed was approximately 0.6 g per experiment. Four chromatograms were recorded for each reaction temperature.

The surface morphologies of representative samples were examined by SEM FEI Quanta 3d FEG (FEI, Hillsboro, OR, USA), with detectors of: SE and BSE, and with EDS equipment.

The phase structure of the surface products after catalysis was examined by XRD using a Seifert 3000 diffractometer (Seifert GmbH, Ahrensburg, Germany) with CoKα radiation at operating parameters of 30 mA, 50 kV and a step size of 0.02° per 3 s.

## 4. Conclusions

With our experiments we showed that thin foils of Ni_3_Al intermetallic alloy are a promising catalyst for cyclohexane decomposition. The results obtained are in line with research conducted for catalytic decomposition of methanol [4,5,6,19] on the same catalyst. In particular, the major findings of the present work can be listed as follows:

(1) The conversion rate of cyclohexane is approximately 99% ± 1% at 600 °C and 87% ± 4% at 500 °C, what is a promising result.
(2) The SEM analyses of the deposit formed on the Ni_3_Al foil after decomposition revealed existence of CNFs, in both platelet and tubular morphologies.
(3) Nickel nanoparticles were found in the CNFs formed during the reaction. The Ni-ended CNFs were obtained during the catalytic decomposition of cyclohexane on the Ni_3_Al intermetallic foil.

## References

[1] Sikka V.K., Deevi S.C., Viswanathan S., Swindeman R.W., Santella M.L. (2000). Advances in processing of Ni_3_Al-based intermetallics and applications. Intermetallics.

[2] Deevi S.C., Sikka V.K. (1996). Nickel and iron aluminides: An overview on properties, processing, and applications. Intermetallics.

[3] Deevi S.C., Sikka V.K., Liu C.T. (1997). Processing, properties, and applications of nickel and iron aluminides. Prog. Mater. Sci..

[4] Kim S.H., Oh M.H., Kishida K., Hirano T., Wee D.M. (2004). Cyclic oxidation behavior and recrystallization of cold-rolled Ni_3_Al foils. Mater. Lett..

[5] Chun D.H., Xu Y., Demura M., Kishida K., Wee D.M., Hirano T. (2006). Spontaneous catalytic activation of Ni_3_Al thin foils in methanol decomposition. J. Catal..

[6] Chun D.H., Xu Y., Demura M., Kishida K., Oh M.H., Hirano T., Wee D.M. (2006). Catalytic Properties of Ni_3_Al Foils for Methanol Decomposition. Catal. Lett..

[7] Arkatova L.A., Pakhnutov O.V., Shmakov A.N., Naiborodenko Y.S., Kasatsky N.G. (2011). Pt-implanted intermetallides as the catalysts for CH_4_–CO_2_ reforming. Catal. Today.

[8] Bojar Z., Jóźwik P., Bystrzycki J. (2006). Tensile properties and fracture behavior of nanocrystalline Ni_3_Al intermetallic foil. Scr. Mater..

[9] Borodians’ka H., Demura M., Kishida K., Hirano T. (2002). Fabrication of thin foils of binary Ni-Al γ/γ′ two-phase alloys by cold rolling. Intermetallics.

[10] Demura M., Kishida K., Suga Y., Takanashi M., Hirano T. (2002). Fabrication of thin Ni_3_Al foils by cold rolling. Scr. Mater..

[11] Li Y.F., Guo J.T., Zhou L.Z., Ye H.Q. (2004). Effect of recrystallization on room-temperature mechanical properties of Zr-doped Ni_3_Al alloy. Mater. Lett..

[12] Jóźwik P., Bojar Z., Winiarek P. (2010). Catalytic activity of Ni_3_Al foils in decomposition of selected chemical compounds. Mater. Eng..

[13] Liu J., Chai Y., Liu B., Wu Y., Li X., Tang Z., Liu Y., Liu C. (2014). The catalytic performance of Ni_2_P/Al_2_O_3_ catalyst in comparison with Ni/Al_2_O_3_ catalyst in dehydrogenation of cyclohexane. Appl. Catal. A General..

[14] Zhou J., Yang X., Wang Y., Chen W. (2014). An efficient oxidation of cyclohexane over Au@TiO_2_/MCM-41 catalyst prepared by photocatalytic reduction method using molecular oxygen as oxidant. Catal. Commun..

[15] Cao Y., Luo X., Yu H., Peng F., Wang H., Ning G. (2013). sp2- and sp3-hybridized carbon materials as catalysts for aerobic oxidation of cyclohexane. Catal. Sci. Technol..

[16] Tanaka A., Seong-Ho Y., Mochida I. (2004). Preparation of highly crystalline nanofibers on Fe and Fe–Ni catalysts with a variety of graphene plane alignments. Carbon.

[17] Rodriguez N.M., Chambers A., Baker R.T.K. (1995). Catalytic engineering of carbon nanostructures. Langmuir.

[18] Martin-Gullon I., Vera J., Conesa J.A., Gonzalez J.L., Merino C. (2006). Differences between carbon nanofibers produced using Fe and Ni catalysts in a floating catalyst reactor. Carbon.

[19] Michalska-Domańska M., Norek M., Jóźwik P., Jankiewicz B., Stępniowski W.J., Bojar Z. (2014). Catalytic stability and surface analysis of microcrystalline Ni_3_Al thin foils in methanol decomposition. Appl. Surf. Sci..

